# Meiotic errors followed by two parallel postzygotic trisomy rescue events are a frequent cause of constitutional segmental mosaicism

**DOI:** 10.1186/1755-8166-5-19

**Published:** 2012-04-10

**Authors:** Caroline Robberecht, Thierry Voet, Gülen E Utine, Albert Schinzel, Nicole de Leeuw, Jean-Pierre Fryns, Joris Vermeesch

**Affiliations:** 1 Department of Human Genetics, Catholic University Leuven, Leuven, Belgium; 2 Department of Pediatrics, Division of Genetics, Hacettepe University, Ankara, Turkey; 3 Institute of Medical Genetics, University of Zürich, Zürich, Switzerland; 4 Department of Human Genetics, Radboud University Nijmegen Medical Centre, Nijmegen, the Netherlands

**Keywords:** Segmental aneusomy, Duplication, Deletion, Somatic mosaicism, Constitutional, Mechanism, Prezygotic, Mitotic, Postzygotic, Meiotic

## Abstract

Structural copy number variation (CNV) is a frequent cause of human variation and disease. Evidence is mounting that somatic acquired CNVs are prevalent, with mosaicisms of large segmental CNVs in blood found in up to one percent of both the healthy and patient populations. It is generally accepted that such constitutional mosaicisms are derived from postzygotic somatic mutations. However, few studies have tested this assumption. Here we determined the origin of CNVs which coexist with a normal cell line in nine individuals. We show that in 2/9 the CNV originated during meiosis. The existence of two cell lines with 46 chromosomes thus resulted from two parallel trisomy rescue events during postzygotic mitoses.

## Background

For decades, knowledge about copy number variation (CNV) in the human genome was limited to microscopically visible changes. Advances in technology have led to the discovery of submicroscopic CNVs, ranging from kilobases to megabases in size and covering up to 13% of the human genome [1,2]. These CNVs can cause recurrent genomic disorders and sporadic disease, or they can represent benign changes found in the healthy population [3,4]. Recent studies have revealed that CNVs are not only polymorphic between unrelated individuals, but also form a frequent source of somatic variation [5,6].

Chromosomal mosaicism is defined as the coexistence of two or more chromosomally different cell lines in an organism which developed from a single zygote. The majority of those mosaicisms are aneuploidies. Several studies investigating *in vitro *fertilized embryos at the preimplantation stage demonstrated a very high number of chromosomal mosaicisms in early human embryos [7-9]. While many of these embryos will not reach the stage of implantation, some do continue to develop leading to fetal mosaicisms, confined placental mosaicism or mosaic infants. Postnatally, mosaicism is detected in 0.4-1% of patients referred for genetic diagnostic screening [10-12]. A recent study revealed that mosaic aberrations are present in about 0.8% of phenotypically normal adults [13]. In addition, mosaicism appears to be variable amongst different tissues: chromosomal aneuploidies were detected in approximately 10% of normal human brain cells [14].

Segmental aneuploidies make up a significant part of mosaic chromosome anomalies. Analysis of several large series of prenatal samples by karyotyping has shown that, of the 0.25-2% mosaic cases that are detected, up to a third comprise segmental imbalances [15,16]. In postnatal clinical diagnosis of patients with developmental anomalies this increases to about half of the mosaic cases [10,17]. The majority of mosaic segmental imbalances are marker chromosomes [16]. A smaller number of cases have been reported to consist of mosaic segmental deletions and/or duplications, ring chromosomes and translocations that have a 46,abnormal/46,normal karyotype. In recent years various case reports have been published [18-36]. The introduction of genome wide aneuploidy detection tools with a higher resolution such as array comparative genomic hybridisation (array CGH) or SNP arrays and the collection of large patient groups have also increased the detection rate of mosaic segmental abnormalities [10,12,17].

For mosaic aberrations, it is intuitively assumed that the rearrangement arose postzygotically. During embryogenesis, a mitotic rearrangement in an otherwise normal diploid embryo results in a second cell line carrying a chromosomal rearrangement. Nevertheless, evidence is mounting that such rearrangements can originate during meiosis. If so, a trisomic zygote carrying the abnormal chromosome has to be rescued twice, in parallel: once loosing the normal and once loosing the abnormal chromosome. Considering that the mitotic error rate is extremely high during early embryogenesis [7-9] and that most cases of mosaic aneuploidy detected in a large cohort of patients were of mitotic origin [11], we reasoned that the latter mechanism might be an important mechanism by which such mosaics arise. In this study we collected nine cases with mosaic structural imbalances to determine their origin and to ascertain whether a meiotic or postzygotic origin might be more prevalent.

## Results

**Figure 1:**
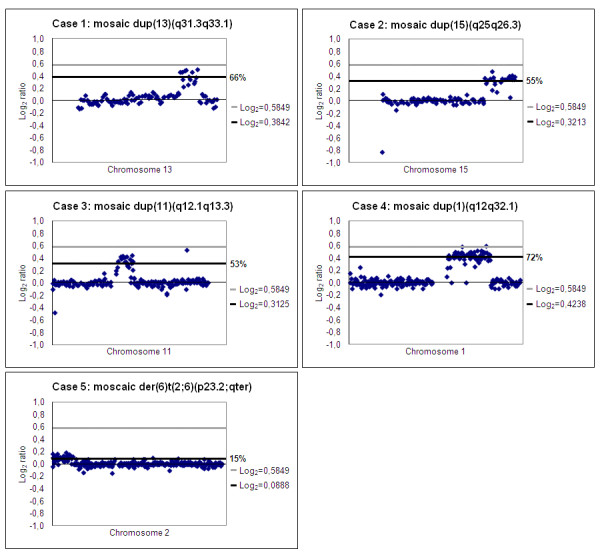
**Microarray profiles of the affected chromosome in cases 1, 2, 3, 4 and 5**. The dots on the X-axis represent the BAC clones ordered from the short-arm telomere to the long-arm telomere. The Y-axis shows log_2 _transformed intensity ratios of the combined dye-swap BAC array experiments (case Cy5/control Cy3). The grey bar indicates the theoretical log_2 _ratio of a non-mosaic duplication, while the thick black bars indicate the individual mosaicism level per case.

**Figure 2:**
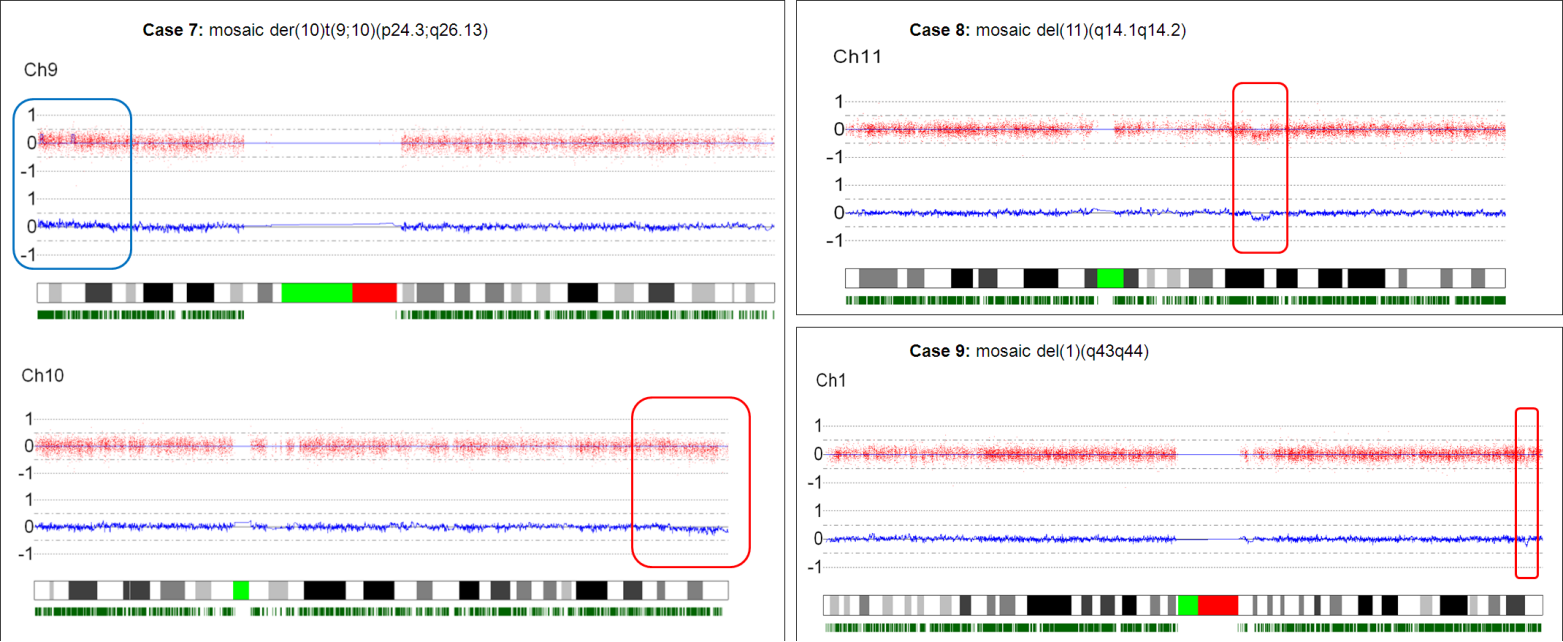
**Microarray profiles of the affected chromosome in cases 7, 8 and 9**. The plots of the aberrant chromosomes obtained with 250 k SNP array analysis are shown with test over reference log2 intensity ratios on the Y-axis plotted against the Mb position from pter to qter on the X-axis. Each red dot represents the test over reference ratio for an individual SNP and the blue line the average test over reference ratio per 10 SNP values.

All nine samples contained two different cell lines, each with 46 chromosomes: one normal diploid cell line and a second cell line carrying a chromosomal rearrangement. In five cases the mosaicism was detected in blood lymphocytes, in three cases (case 4, 8 and 9) in amniocytes and in one case (case 6) in chorionic villi. In addition to the mosaicism present in the white blood cells, the mosaicism was confirmed in buccal cells in cases 7 and 9. Conventional karyotyping was carried out in five cases and detected the mosaicism in four out of five. Microarrays were performed on DNA from 8/9 cases to identify or confirm the segmental aneuploidy, determine its size and rule out any additional chromosomal imbalances (Figures 1 and 2). The array intensity ratios indicated duplications in approximately 66, 55, 53, 72 and 15% of the cells in patients 1, 2, 3, 4 and 5 respectively. The array intensity ratios indicated the presence of segmental deletions in approximately 20 and 30% of the cells in samples 8 and 9. In two samples, 6 and 7, both deletions and duplications were identified by karyotyping (case 6) or array CGH (case 7) in respectively 50 and 20% of the cells. Conventional karyotyping showed those imbalances to be unbalanced translocations. FISH confirmed the mosaic segmental imbalances in six samples. FISH also determined that the duplication in cases 1 and 4 was not translocated to another chromosome and that cases 5 and 7 carry unbalanced translocations. The karyotypes are presented in Table 1. The degree of mosaicism was estimated based on the intensity ratios of the targets located within the segmental aneuploidy or on data from FISH analysis. The segment sizes and estimated relative presence of the segmental aneuploidy range between 1 and 124 Mb and an overview is shown in Table 1. Chromosome analysis of the parents, including FISH for submicroscopic aberrations, was normal for all.

**Table 1:** Combined karyotypes after conventional and molecular cytogenetic analyses

case	Karyotype (ISCN 2009; Mb positions mapped in hg19)	% mos	del/dup size
1	46,XX,dup(13)(q31.3q33.1)dn/46,XX.arr 13q31.3q33.1 (RP11-319 L6-RP11-564 N10)x2 ~3	66%	11.11 Mb

2	46,XX,dup(15)(q25q26.3)dn/46,XX.arr 15q25.2q26.3 (RP11-365 F16-CTB-154P1)2 ~3	55%	18.25 Mb

3	46,XY,dup(11)(q12.1q13.3)dn/46,XY.arr 11q12.1q13.3 (RP11-131 J4-RP11-804 L21)2 ~3	53%	14.55 Mb

4	46,XY,dup(1)(q12q32.1)dn/46,XY.arr 1q12q32.1 (RP11-417 J8-RP11-383 G10)2 ~3	72%	62.72 Mb

5	46,XX,der(6)t(2;6)(p23.2;qter)dn/46,XX.arr 2p25.3p23.2 (GS1-68 F18-RP11-328 L16)2 ~3	15%	29.36 Mb

6	46,XY,der(20)t(1;20)(10q;10p)dn/46,XY *	50%	25.56 Mb/124.2 Mb

7	46,XX,der(10)t(9;10)(p23;q26.13)dn/46,XX.arr 9p24.3p23(40,910-13,575,891)x2 ~3, 10q26.13q26.3 (124,007,108-135,422,505)x1 ~2	20%	13.53 Mb/11.31 Mb

8	46,XX.ish del(11)(q14.1q14.2)(RP11-118 L16-,RP11-157B22-)dn[21/50].arr 11q14.1q14.2(83,122,844-86,794,856)x1 ~2	20%	3.7 Mb

9	46,XX.ish del(1)(q43q44)(RP11-113O11-,RP11-370 K11-)dn[25/50].arr 1q43q44(242,916,876-243,920,382)x1 ~2	30%	~1 Mb

*Insufficient DNA for array analysis

**Figure 3:**
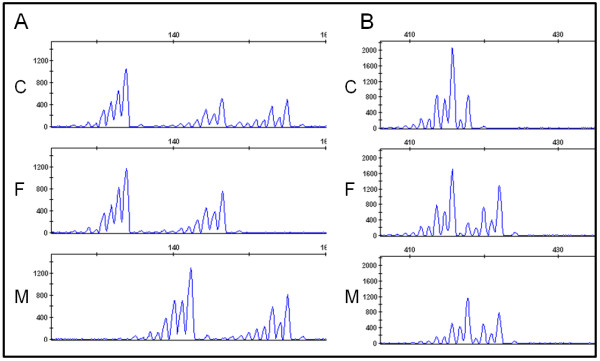
**Short tandem repeat analysis on DNA of the patient and parents**. A) Case 1 has three different alleles for marker D13S128 corresponding to two different paternal alleles and one maternal allele. B) At marker D13S129 case 1 has two identical paternal alleles and one maternal allele. This indicates the paternal origin of the duplication in chromosome 13 and suggests a cross-over occurred between the paternal chromosomes 13 followed by a meiosis II non-disjunction and an unequal sisterchromatid exchange. C: case, F: father, M: mother.

**Figure 4:**
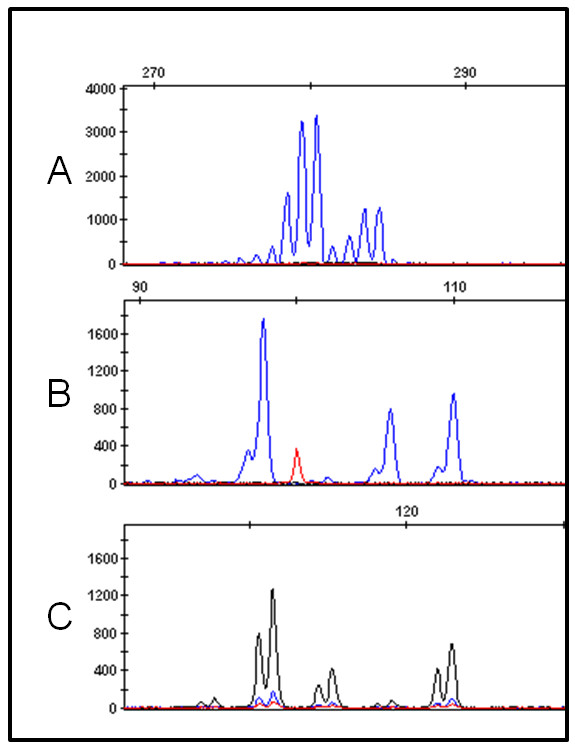
**Short tandem repeat analysis on fetal DNA of case 6**. Case 6 shows two alleles with a 2:1 ratio for marker D1S1595 (A) and three different alleles for markers D1S1653 (B) and D1S1171 (C). This suggests a meiosis I origin for the duplicated segment of the unbalanced translocation.

**Figure 5:**
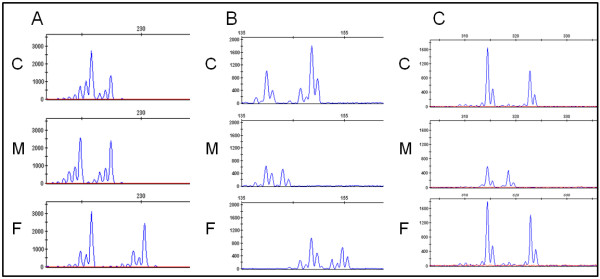
**Short tandem repeat analysis on DNA of cases 2, 4 and 5 and parental DNA**. A) Case 2 has a difference in peak ratios in marker D15S130 corresponding to two identical paternal alleles and one maternal allele. B) Case 4 demonstrates a 2:1 peak ratio in marker D1S2635 corresponding to two identical paternal alleles and one maternal allele. C) Case 5 shows a duplication of the maternal allele in marker D2S1780. C: case, F: father, M: mother.

To determine whether the initial rearrangement occurred during meiosis or during postzygotic mitosis, polymorphic marker analysis or SNP array analysis was performed. For patients 1 to 6, carrying segmental duplications, markers were selected within and flanking the duplicated region. If the duplication arose during meiosis, three alleles might be observed. If only two alleles are detected, there are two possible explanations: either the duplication arose postzygotically or the duplication arose during meiosis and the segmental duplication is one of both sister chromatids present during meiosis I or II. Results of the short tandem repeat (STR) analyses are shown in Figures 3, 4 and 5 and a summary is presented in Table 2.

**Table 2:** Results of the STR marker analysis

case 1	marker	location	F/C/M
	D13S232	13q12.12	ac/ac/ab
	**D13S129**	**13q31.3**	ac/**aab**/bc
	**D13S128**	**13q32.2**	ac/**acd**/bd
	**D13S770**	**13q32.3**	ab/aab/ab
	**D13S779**	**13q32.3**	ab/aab/ac
	D13S1315	13q34	ac/bc/ab
	D13S285	13q34	b/bc/ac

**case 2**	**marker**	**location**	**F/C/M**

	D15S165	15q13.2-q13.3	ab/ab/ac
	D15S222	15q21.1	ab/ab/ab
	**D15S116**	**15q26.1**	bc/**bbc**/ac
	**D15S127**	**15q26.1**	cd/**bdd**/ab
	**D15S158**	**15q26.1**	ac/**bcc**/ab
	**D15S130**	**15q26.2**	bd/**bbc**/ac

**case 3**	**marker**	**Location**	**F/C/M**

	**D11S1357**	**11q12.1**	b/b/ab
	**D11S480**	**11q12.3**	bd/**bbc**/ac
	**D11S913**	**11q13.1-q13.2**	?/**aac**/bc
	**D11S4095**	**11q13.3**	bc/**abb**/ad
	D11S1314	11q13.4	?/bc/ab
	D11S916	11q13.4	?/a/a
	D11S1339	11q22.1-q22.2	?/ab/ac
	D11S4111	11q23.3	?/bc/ab

**case 4**	**marker**	**location**	**F/C/M**

	**D1S1595**	**1q22**	ac/c/bc
	**D1S1653**	**1q23.1**	?/b/ab
	**D1S2635**	**1q23.2**	cd/**acc**/ab
	**D1S2705**	**1q23.3**	ac/**aab**/b
	**D1S212**	**1q25.2**	ac/**bcc**/bd
	**D1S1171**	**1q32.1**	?/a/ab
	D1S2631	1q42.13	a/ab/ab

**case 5**	**marker**	**location**	**F/C/M**

	**D2S2268**	**2p25.3**	b/b/ab
	**D2S2245**	**2p25.3**	ac/**bbc**/bc
	**D2S319**	**2p25.3**	ab/**bcc**/bc
	**D2S1780**	**2p25.3**	ac/**aac**/ab

**case 6**	**marker**	**location**	

	**D1S1595**	**1q22**	aab
	**D1S1653**	**1q23.1**	**abc**
	**D1S2635**	**1q23.2**	ab
	**D1S2705**	**1q23.3**	a
	**D1S212**	**1q25.2**	ab
	**D1S1171**	**1q32.1**	**abc**

**case 6**	**marker**	**location**	

	**D20S117**	**20p13**	ab

Results of the STR marker analysis. Highlighted markers lie within the duplicated or deleted regions. Informative marker results are underlined and highlighted. F: father, C: case, M: mother.

For two samples, three different alleles were detected within the duplicated region indicating a meiotic origin. Polymorphic marker analysis of sample 1 revealed the presence of three different alleles for marker D13S128 (Figure 3A). Two of the alleles were inherited from the father demonstrating the paternal origin of the segmental aneusomy. At marker D13S129, still within the duplication, two alleles with a 2:1 intensity ratio were detected, including only one of the two paternal alleles (Figure 3B). The remaining markers within the duplication were not informative. In case 6, carrier of an unbalanced translocation, two markers (D1S1653 and D1S1171) spread throughout the duplicated 1q region showed three different alleles (Figure 4). The other markers on 1q had either one or two alleles. Marker D20S117 in the deleted 20p region confirmed the deletion, with a 1:2 reduced peak intensity for one of both alleles.

For the other cases, only two different alleles were identified within the duplication region. Genotyping of cases 2 to 5 within the region of duplication showed in each case only two alleles. One of both alleles clearly had a higher intensity with about a 2:1 ratio (Figure 5). For sample 5, in which array CGH revealed that the rearrangement is only present in 10 to 20% (log_2 _value of 0.0888) of all cells, these peak intensity differences were still present, but to a lesser extent.

**Figure 6:**
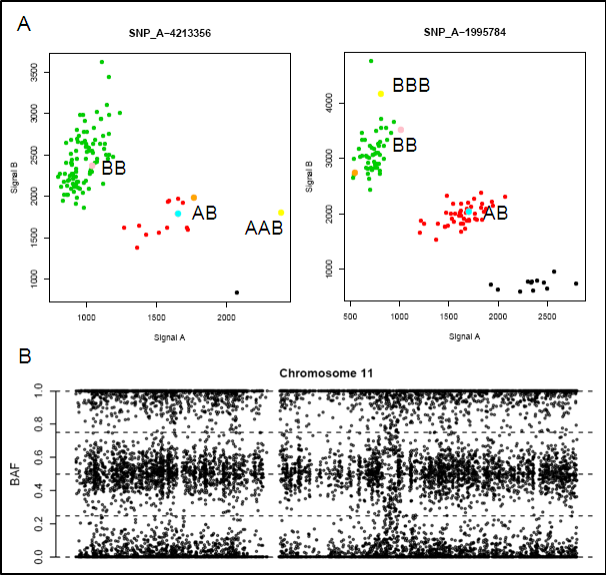
**SNP array analysis of cases 7 and 8**. A) SNP cluster plots of individual SNPs. Green, red and black dots represent controls with a BB, AB and AA genotype respectively. The pink and blue dots represent the genotypes of the mother and father. The yellow dots indicate the genotype of case 7 in cells with the duplication, while the orange dots give the genotype of the normal cells of the fetus. The SNPs shown are located within the paternal duplication. The plots indicate that paternal heterozygous SNPs in the duplicated region do not show transmission of both paternal alleles, but instead revealed a duplication of one of the paternal alleles. This suggests the duplication originated during postzygotic mitosis or meiosis II. B) The B allele frequency graph of chromosome 11 shows abnormal heterozygous values without complete loss of heterozygosity in the deleted segment in case 8. No additional haplotypes are found in the regions surrounding the deletion. This rules out the possibility of a trisomy rescue and indicates the deletion originated during postzygotic mitosis or meiosis II.

For patients 7 to 9 with mosaic segmental deletions, single nucleotide polymorphisms were analyzed by 250 K Affymetrix SNP arrays. To determine the origin of the mosaic unbalanced translocation in case 7, genotyped by Affymetrix SNP arrays, the following strategy was followed: the A and B allele ratios of individual SNPs within the segmental mosaic duplication and deletion were determined for those SNPs that were homozygous but with a different allele in both parents. Analysis of the A and B allele ratios transmitted from parental homozygous AA and BB SNPs showed 77 SNPs to fulfill this criteria in the duplication and 51 SNPs in the deletion and demonstrated a paternal origin for both the deleted and the duplicated segments of the unbalanced translocation. Subsequently, the A and B allele ratios of SNPs in the duplicated region that were paternal heterozygous but maternal homozygous were interrogated. Analysis of the SNPs did not show transmission of both paternal alleles. Only a single duplicated allele from the father was detected (Figure 6A).

To determine the origin of the deletion in cases 8 and 9, again for the SNPs that were homozygous but with a different allele in both parents in the deletion, the A and B allele ratios were determined. The analysis showed that the signal of the paternal allele was reduced in case 8 and that a maternal allele was removed in case 9. To search for evidence of a potential meiotic event, the rest of the chromosome was screened for the presence of two paternal (case 8) or two maternal alleles (case 9). Hereto, the B-allele frequencies of SNPs on the affected chromosome, but outside the somatic CNV region, were evaluated for additional haplotypes. If the deletion resulted from a meiotic event, altered B-allele frequencies would be observed, with additional values between 0 and 0.5 as well as between 0.5 and 1. No such haplotypes were detected in the regions surrounding the deletion (Figure 6B).

The combination of a normal and an aberrant cell line in these cases could, aside from mosaicism, also result from chimerism: the fusion of two cell lineages from separate fertilization events. In samples 1 to 6, the presence of chimerism was evaluated by testing four STR markers spread over different chromosomes not involved in the aberrant region. in case of a mosaic all autosomal genotypes outside the duplicated region are expected to only contain two alleles (1 maternal & 1 paternal). For chimeras, some loci would have three or four alleles, or a skewed dosage of two alleles [37]. None of the samples showed more than two alleles on any of the markers, confirming the mosaic status of the aberration (data not shown). In cases 7 to 9, the two different genotypes present in case of chimerism would be seen as aberrant B-allele frequencies in all autosomes, with an altered ratio between the two haplotypes. No alterations in the B-allele frequency were seen in the unaffected chromosomes, thereby ruling out the possibility of chimerism.

## Discussion

**Figure 7:**
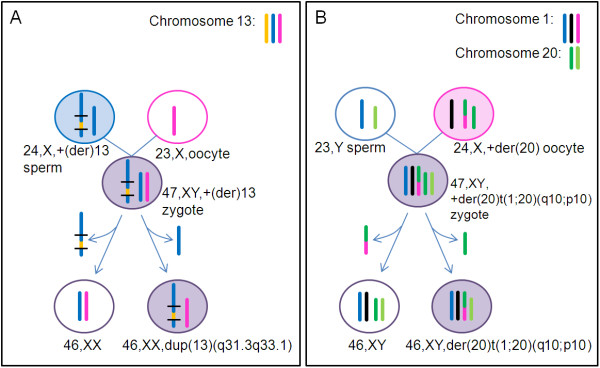
**Schematic representation of the events leading to the mosaicism in cases 1 and 6**. A) In case 1, there was likely a recombination between the two paternal homologous chromosomes 13, followed by a duplication of part of the recombined chromosome. A subsequent non-disjunction resulted in a trisomic zygote, that lead to a normal and an abnormal cell line through two trisomy rescue events. B) The mosaicism in case 6 presumably originated from a translocation between chromosome 1 and chromosome 20 during meiosis I, with segregation of a normal and a derivative chromosome 20 to the zygote. This results in a zygote which is trisomic for chromosome 20p and 1q and could lead to the mosaicism seen in this case by trisomy rescue of the derivative chromosome (normal cells) and trisomy rescue of a normal chromosome 20 (abnormal cells).

Somatic chromosomal mosaicisms are thought to arise from postzygotic somatic mutations. Here, we show that in two out of nine cases (>15%) the rearrangements occurred preconception. Cases 1 and 6 showed markers with three different alleles in the duplicated region. The most parsimonious explanation is that both aberrations originated from a meiotic segregation error resulting in a trisomic conception, followed by two parallel trisomy rescue events during the successive mitotic divisions (Figure 7). In the other seven cases (pt 2-5 & pt 7-9), the duplication was derived from the chromosome with the same parental allele as the transmitted chromosome in the normal cell line or the deletion was on the chromosome with the same parental allele as the normal chromosome in the normal cell line. In these cases the copy variation likely occurred during postzygotic cell divisions. Nevertheless, those rearrangements could also have occurred during meiosis II. Hence, the percentage of mosaic segmental imbalances generated during meiosis by two separate postzygotic trisomy rescue events could be higher than 15%. The occurrence of multiple postzygotic trisomy rescue events may appear to be unexpected. However, a high frequency of mosaic trisomies and monosomies is reported to occur during cleavage stage in *in vitro *fertilized embryos [7-9]. The observation that two independent trisomy rescue events can underlie those mosaicisms is further testimony of this high mutational burden during early embryogenesis. Postzygotic trisomy rescue events are also frequently observed in patients with imprinting diseases, including Prader-Willi syndrome and Silver-Russell syndrome. Such patients may be fully disomic with complete uniparental disomy (UPD) or be mosaic with a trisomic and a disomic (UPD) cell line [11,38-40].

While the origin of mosaic segmental CNVs has only rarely been investigated, two case reports support our finding that pre-zygotic rearrangements followed by multiple postzygotic trisomy rescue events can underlie segmental mosaicisms. Blouin et al. detected a mosaic *de novo *direct tandem duplication of 21q11.2q22.2 that resulted from a meiosis I crossover followed by an unequal sister chromatid exchange and two trisomy rescue events, similar to our case 1. The mosaic *de novo *unbalanced translocation with partial trisomy 16p and maternal UPD 16 reported by Schinzel et al. likely originated from a maternal trisomy 16 (MI), a subsequent postzygotic translocation of the paternal 16p segment and finally loss of the paternal chromosomes 16 in the translocated and normal cell lines [18,32]. Even after combining our results with previous mosaic segmental cases the total study population remains small, but future studies may be able to provide additional evidence for the high frequency of multiple independent trisomy rescue events.

## Conclusions

In conclusion, we demonstrated that in 2/9 cases, the detected mosaic segmental aberrations resulted from meiotic errors followed by multiple parallel trisomy rescue events and not from postzygotic mitotic changes. In addition, we show that the use of sensitive array technology is especially suited for the detection of mosaicism, which is clinically relevant to a patient's diagnosis.

## Methods

### Patient selection

Five cases were referred for cytogenetic/molecular investigations after clinical evaluation for dysmorphic features, whereas cases 4, 6, 8 and 9 were ascertained during prenatal diagnosis (CVS and/or amniocentesis).

### Cytogenetic Analysis

Cytogenetic analyses were performed on GTG-banded metaphase chromosomes from PHA-stimulated peripheral blood lymphocytes or from CVS cells or amniocytes after standard culture and chromosome preparation protocols. Twenty metaphases with a resolution of 550 bands per haploid genome were karyotyped (ISCN 2009).

BAC microarray analysis was performed as described elsewhere [41]. Briefly, CodeLink Bioarray System slides (Amersham Biosciences, Chalfont St.Giles, UK) were used for array construction using a 1 Mb clone set of 3 683 BAC and PAC clones. Genomic DNA from the index patient and two other patients was labelled in Cy3 and Cy5, respectively (Amersham Biosciences, Chalfont St.Giles, UK) with random prime labelling (Bioprime array CGH, Invitrogen, Sunnydale, CA) and hybridized in a 3-way experiment. Hybridization and post-hybridization washing steps were performed as previously described [41]. Slides were scanned at 532 nm (Cy3) and 635 nm (Cy5) using a GenePix 4000B microarray scanner (Axon Instruments, Union City, CA) with the software GenePixPro 6.0. Data analysis was performed with Excel (Microsoft Inc.; Diegem, Belgium) as described [41]. The percentage of mosaicism was calculated by dividing the mean log2 ratio of the BAC clones in the duplicated region by the log2 of a non-mosaic aberration (0.5849).

SNP array analysis was performed on Affymetrix GeneChip^® ^Human Mapping 250 K NspI arrays (Affymetrix, Inc., Santa Clara CA, USA) containing 25-mer oligonucleotides representing a total of 262 264 SNPs. DNA digestion, labeling and hybridization were performed according to the manufacturer's protocol. After hybridization, arrays were washed and stained on the Affymetrix GeneChip fluidics station 450 and scanned using the Affymetrix GeneChip^® ^Scanner 3000 7 G. Data analysis was performed using Copy Number Analyzer for GeneChip version 2.0 (CNAG 2.0) [42] and Genotyping Console™ Software (Affymetrix, Inc.).

### Fluorescent in situ hybridization

To confirm aberrations found by microarray, FISH was performed with BAC probes as described [43], using an optical fluorescence microscope (Olympus U-SPT, BX51, Japan) with selective filters equipped with CytoVision^® ^software (Applied Imaging, Genetix, Gateshead, UK).

### SNP cluster determination

For determining the parental origin of the DNA copy number aberrations by a SNP-cluster strategy, normalized SNP A and B allele intensities as well as SNP genotype calls were computed using Affymetrix power tools (APT-1.10.1) in combination with the Birdseed command [44]. Besides the described trio datasets, in-house produced Affymetrix 250 K SNP NspI data from 102 additional DNA samples were co-processed for accurate SNP cluster and genotype determination. Subsequently, the probe intensities and genotype calls for SNPs within the regions of interest were retrieved for all DNA samples and interpreted by custom R-code [45] in which patient and parental SNP-probe intensities are visualised in the Birdseed SNP clusters from the 102 individuals.

### B-allele frequency plots

To generate the B-allele frequency (BAF) plots, copy number analysis of the SNP array data was performed using the Affymetrix power tools BRLMM, a modified version of RLMM [46] and the pennCNV [47] algorithm. These data were subsequently further interpreted and visualized in BAF and logR ratio plots by the genoCN algorithm [48].

### Polymorphic microsatellite marker analysis

Polymorphic microsatellite markers made up of dinucleotide (CA)n repeats (short tandem repeats or STRs) were selected from the Ensembl [49] and UCSC [50] genome browser databases and the oligonucleotides were purchased from Eurogentec S.A. (Seraing, Belgium) and Pharmacia Biotech (Uppsala, Sweden). Genomic DNA was extracted from peripheral blood leukocytes, amniocytes or CVS cells of the patients and their parents according to standard procedures and CA repeats spaced along the involved chromosomes were amplified by polymerase chain reaction (PCR). DNA extracted from skin fibroblasts was also included in the analysis in case the mosaicism was encountered in fibroblasts.

After PCR amplification of the STRs, fragments were analyzed by gel electrophoresis. Automated fragment sizing was performed on the ABI Prism 3100 Genetic Analyzer (Applied Biosystems, Nieuwerkerk a/d Ijssel, the Netherlands), using software GeneScan or Genemapper v4.0 (Applied Biosystems, Nieuwerkerk a/d Ijssel, The Netherlands).

## Competing interests

The authors declare that they have no competing interests.

## Authors' contributions

CR carried out the molecular genetic studies, analyzed the results and drafted the manuscript. TV performed the SNP cluster determination and helped to draft the manuscript. SA participated in the design of the study and helped to draft the manuscript. GU participated in the molecular genetic studies and the results analysis. DLN carried out the SNP arrays and analyzed the data. JV conceived of the study, participated in its design and coordination and helped to draft the manuscript. All authors read and approved the final manuscript.
